# Study-related wellbeing, behavior, and attitudes of university students in the Netherlands during emergency remote teaching in the context of COVID-19: A longitudinal study

**DOI:** 10.3389/fpsyg.2022.1056983

**Published:** 2022-12-06

**Authors:** Manja Vollmann, Renée A. Scheepers, Anna P. Nieboer, Femke Hilverda

**Affiliations:** Department of Socio-Medical Sciences, Erasmus School of Health Policy & Management, Erasmus University Rotterdam, Rotterdam, Netherlands

**Keywords:** emergency remote teaching, academic burnout, study engagement, study effort, education satisfaction, online self-efficacy, attitudes toward online education

## Abstract

**Introduction:**

During the COVID-19 pandemic, emergency remote teaching was implemented at all conventional Dutch universities; however, the degree of limitations in on-campus teaching and learning varied during the pandemic dependent on the strictness of the measures. In the present study, it will be investigated how study-related experiences of university students changed in the face of varying limitations in on-campus teaching and learning.

**Methods:**

The study had a longitudinal natural experiment design with three points of measurement during the academic year 2020–2021: November–December 2020 (t1; campuses partially open), March 2021 (t2; campuses fully closed) and June–July 2021 (t3; campuses partially open). In total, 680 Dutch university students (65.9% female; age: *M* = 21 years, *SD* = 2.06) filled in online surveys measuring study-related wellbeing (academic burnout and study-engagement), study-related behavior (study effort), and study-related attitudes (education satisfaction, online self-efficacy, and attitudes toward online education).

**Results:**

Overall, students reported moderate levels of academic burnout, study engagement, study effort, education satisfaction, and online self-efficacy; their attitudes toward online education were rather negative. Students’ study-related wellbeing and education satisfaction decreased in the period when on-campus teaching and learning was impossible (t2) compared to periods in which on-campus teaching and learning was possible at a low level with several restrictions (t1 and t3). Students’ attitudes toward online education and online self-efficacy slightly increased at the end of the academic year (t3); however, the attitudes toward online education remained negative.

**Discussion:**

The findings indicate that students’ academic burnout, study engagement, and education satisfaction varied over the course of the academic year in the context of changing limitations in on-campus teaching and learning. To facilitate positive study-related experiences, universities are advised to offer as much on-campus education as possible in times of pandemics.

## Introduction

During the COVID-19 pandemic, most universities worldwide had to (partially) close their campuses due to preventive measures imposed by governments to mitigate the spread of the coronavirus. Traditional on-campus or face-to face teaching and learning with physical class meetings was severely limited or even impossible (Marinoni et al., 2020; National Institute for Public Health and the Environment, 2022). To continue education, universities rapidly implemented emergency remote teaching (ERT; Hodges et al., 2020) mainly consisting of recorded or written presentations and instructions, online discussion boards, and classes *via* collaboration and video conferencing platforms (Aristovnik et al., 2020; Mseleku, 2020). ERT was implemented all through the pandemic; however, the degree of limitations in on-campus teaching and learning varied during the pandemic dependent on the strictness of the measures (Marinoni et al., 2020; National Institute for Public Health and the Environment, 2022).

The pandemic-induced changes in university education together with the changes in everyday life had a detrimental impact on students’ overall mental health. In particular, several longitudinal studies starting before the pandemic revealed significant increases in the severity and prevalence of depression, anxiety, stress, and loneliness among university students after the onset of the pandemic and its measures compared to the pre-pandemic period (Bu et al., 2020; Elmer et al., 2020; Huckins et al., 2020; Li et al., 2020; Saraswathi et al., 2020; Deng et al., 2021; Fruehwirth et al., 2021; Meda et al., 2021; Savage et al., 2021; Werner et al., 2021; Zimmermann et al., 2021). Additionally, longitudinal studies conducted during the pandemic indicate that, after the initial decline in mental health after the onset of the pandemic and the introduction of ERT, the mental health of students remained relatively stable at a lower level (Schindler et al., 2021; Weber et al., 2022) or varied dependent on the strictness of the measures (Amendola et al., 2021; Meda et al., 2021).

Much less is known, however, about the impact of the limitations in on-campus teaching and learning specifically on students’ study-related experiences, including study-related wellbeing (i.e., academic burnout, study engagement), study-related behaviors (i.e., study effort), and study-related attitudes (i.e., education satisfaction, online self-efficacy, attitudes toward online education). Investigating changes in these study-related experiences during the pandemic is important as previous research showed that these experiences are in general closely related to students’ academic performances and achievements (Upadyaya and Salmela-Aro, 2013; Chang et al., 2014; Schneider and Preckel, 2017; Lei et al., 2018; Madigan and Curran, 2020; Pu et al., 2020; Gopal et al., 2021).

### Study-related experiences during the pandemic

The few studies investigating the impact of the pandemic-induced changes in university education on students’ study-related experiences revealed mixed results dependent on the study design. Longitudinal studies suggest that the introduction of ERT did not compromise students’ study-related wellbeing, study engagement, and (online/internet) self-efficacy (Lin, 2021; Pasion et al., 2021; Schindler et al., 2021; Talsma et al., 2021; Zis et al., 2021; Azzi et al., 2022), although one study found an increase in cynicism, a subdomain of academic burnout, among medical students (Zis et al., 2021). On the other hand, cross-sectional studies that measured retrospectively perceived changes suggest a negative impact of ERT as they showed that students perceived an increase in workload, exhaustion, and worries about study issues and career plans as well as a decrease in study engagement, study effort, and education satisfaction after the introduction of ERT (Aristovnik et al., 2020; Elmer et al., 2020; Daniels et al., 2021; Harries et al., 2021; Walker and Koralesky, 2021; Wester et al., 2021; Lee K. et al., 2021).

During the implementation of ERT throughout the pandemic, students reported moderate levels of academic burnout and study engagement, medium to low levels of education satisfaction, moderate to high levels of online self-efficacy, and neutral to negative attitudes toward online education (Aristovnik et al., 2020; Moreno-Fernandez et al., 2020; Aldhahi et al., 2021; Barzani and Jamil, 2021; Guven Ozdemir and Sonmez, 2021; Hamdan et al., 2021; Heidari et al., 2021; Lin, 2021; Silistraru et al., 2021; Simsek et al., 2021; Sveinsdóttir et al., 2021; Talsma et al., 2021; Teuber et al., 2021; Wang et al., 2021; Lee K. et al., 2021; Ertek et al., 2022; Geçer and Bağci, 2022; Kwan, 2022; Silistraru et al., 2022). However, it is still unclear to what extent students’ study-related experiences changed over the course of the pandemic dependent on the strictness of the measures and consequently dependent on the degree of limitations in traditional on-campus teaching and learning.

### The present study

Due to the above-described lack of research, our primary aim was to investigate whether and how study-related experiences of university students changed during the pandemic in the face of varying limitations in traditional on-campus teaching and learning. Using a longitudinal natural experiment design, Dutch university students reported three times on their study-related experiences during the academic year 2020–2021. ERT was implemented at all conventional Dutch universities as main form of education during the entire academic year; the degree of limitations in traditional on-campus teaching and learning varied during the year due to the strictness of the preventive measures (see Procedure for further details).

Students’ experiences from three domains were considered in the present study, i.e., study-related wellbeing, study-related behavior, and study-related attitudes. First, from the domain of study-related wellbeing, the two key concepts according to the study demands-resources framework (Lesener et al., 2020), namely academic burnout and study engagement, were included. Academic burnout refers to a psychological syndrome characterized by emotional exhaustion because of study demands, a cynical and detached attitude toward one’s study, and perceived academic inefficacy (Schaufeli et al., 2002), while study engagement is defined as a positive affective-cognitive state of mind characterized by high levels of energy, enthusiasm and inspiration, and absorption so that time passes quickly (Schaufeli et al., 2002). Second, from the domain of study-related behavior, study effort, i.e., the extent to which students work hard and put effort into their study (van Herpen et al., 2017), was included. Third, three attitudes toward (online) teaching and learning were examined, namely education satisfaction which refers to the students’ overall satisfaction with the educational offering (Sears et al., 2017), online self-efficacy defined as students’ perceptions on their ability to successfully complete an online course (Shen et al., 2013), and attitudes toward online education in general (Ng, 2012).

Our secondary aim was to systematically explore whether potential changes in students’ study-related experiences were modified by gender and study phase (i.e., enrolled in a bachelor vs. master program). Investigating the role of gender and study phase provides insight into whether certain groups of students were more negatively affected by the limitations in traditional university education and consequently would need more attention and support. As regards gender, previous research showed that the pandemic had a stronger negative impact on the mental health of female students compared to male students (Fruehwirth et al., 2021; Harries et al., 2021; Meda et al., 2021). However, less clear is whether gender is related to study-related experiences during the pandemic as earlier studies revealed contradictory results (Aristovnik et al., 2020; Hamdan et al., 2021; Simsek et al., 2021; Werner et al., 2021; Jezzini-Martinez et al., 2022). Also, little is known about to what extent study phase is associated with study-related experiences during the pandemic and the few empirical findings are heterogeneous. Some studies suggest that undergraduate (bachelor) students were more negatively affected by the introduction of ERT as they found it more difficult to focus during online teaching, were more concerned about their future education, were less satisfied with the provided education, and experienced more academic burnout than graduate (master) students (Aristovnik et al., 2020; Hamdan et al., 2021; Simsek et al., 2021; Sveinsdóttir et al., 2021). Other studies found no (systematic) differences in academic burnout and education satisfaction between students from different study phases (Aldhahi et al., 2021; Zis et al., 2021).

## Materials and methods

### Procedure

This longitudinal study with three points of measurement (t1–t3) was conducted during the academic year 2020–2021 *via* the online survey platform Qualtrics XM (Qualtrics, 2020). Full-time students until the age of 30 years old from all 13 conventional public Dutch universities were eligible to participate. Only a small percentage of students studying at conventional universities is older than 30 years. During the academic year 2020–2021, only 4% of graduates were 30 years or older (CBS, 2022). For this reason, they were excluded from this study. The study followed the principles of the Declaration of Helsinki and participants were treated according to the American Psychological Association ethical standards. The Medical Ethics Review Committee of the Erasmus Medical Center approved the study protocol (#2020–0815).

At t1, participants were recruited online *via* emails sent by two large national student organizations and diverse local student associations to their members as well as *via* postings on social media of universities, student associations, and study-related organizations. After opening the provided link, interested students received detailed information about the aim of the study, the study procedure, the terms of participation, and ethical aspects, such as voluntary participation, anonymized data collection and processing, and data use for scientific purposes. Students willing to participate had to agree to the informed consent before getting access to the survey. After filling in the surveys, participants provided their email address separate from their data. *Via* this email address, participants received a monetary compensation and in due time a personal link for the following survey. In Qualtrics, the option to prevent respondents from taking the survey multiple times was activated and all items were set to be mandatory to avoid missing data. As compensation, participants who fully completed the survey received digital gift vouchers of €10 at t1, €15 at t2, and €25 at t3.

### Measurement points

Data were collected at three time points; after the first 2.5 months of the academic year (t1; 18 November 2020–20 December 2020), halfway through the academic year (t2; 11 March 2021–28 March 2021); and at the end of the academic year (t3; 28 June 2021–11 July 2021). During the whole academic year, ERT was implemented at all conventional Dutch universities as the main form of education, however, the degree of limitations in on-campus teaching and learning varied (National Institute for Public Health and the Environment, 2022).

At t1 and t3, universities were partly open the preceding 2.5 months and 1 month, respectively. On-campus teaching and learning was possible at a low level with several restrictions such as 1.5 m distance, wearing a face mask, and a maximum number of students in classes. Only 36.5% (t1) and 43.5% (t3) of the students reported that their education was exclusively online. At t2, the second national lockdown was in force; universities were fully closed the preceding 2.5 months and no on-campus teaching and learning was permitted (except for internships). The majority of students (70.3%) reported that their education was exclusively online.

While ERT was the main form of education during the whole academic year, there were variations in the percentage of how much of the education took place online. Students reported that on average 87.32% (*SD* = 19.38), 93.28% (*SD* = 18.71), and 84.75% (*SD* = 25.04) of their education took place online during the timespan preceding t1, t2, and t3, respectively. A repeated-measures analysis of variance confirmed that the percentage of online education significantly varied between the three measurement points, *F*(2, 1,358) = 34.59, *p* < 0.001, *η*^2^ = 0.05. Pairwise comparisons implied that the percentage of online education was significantly higher at t2 than at t1 and t3, *p*s < 0.001, while no significant difference was observed between t1 and t3, *p* = 0.06.

### Participants

**Figure 1:**
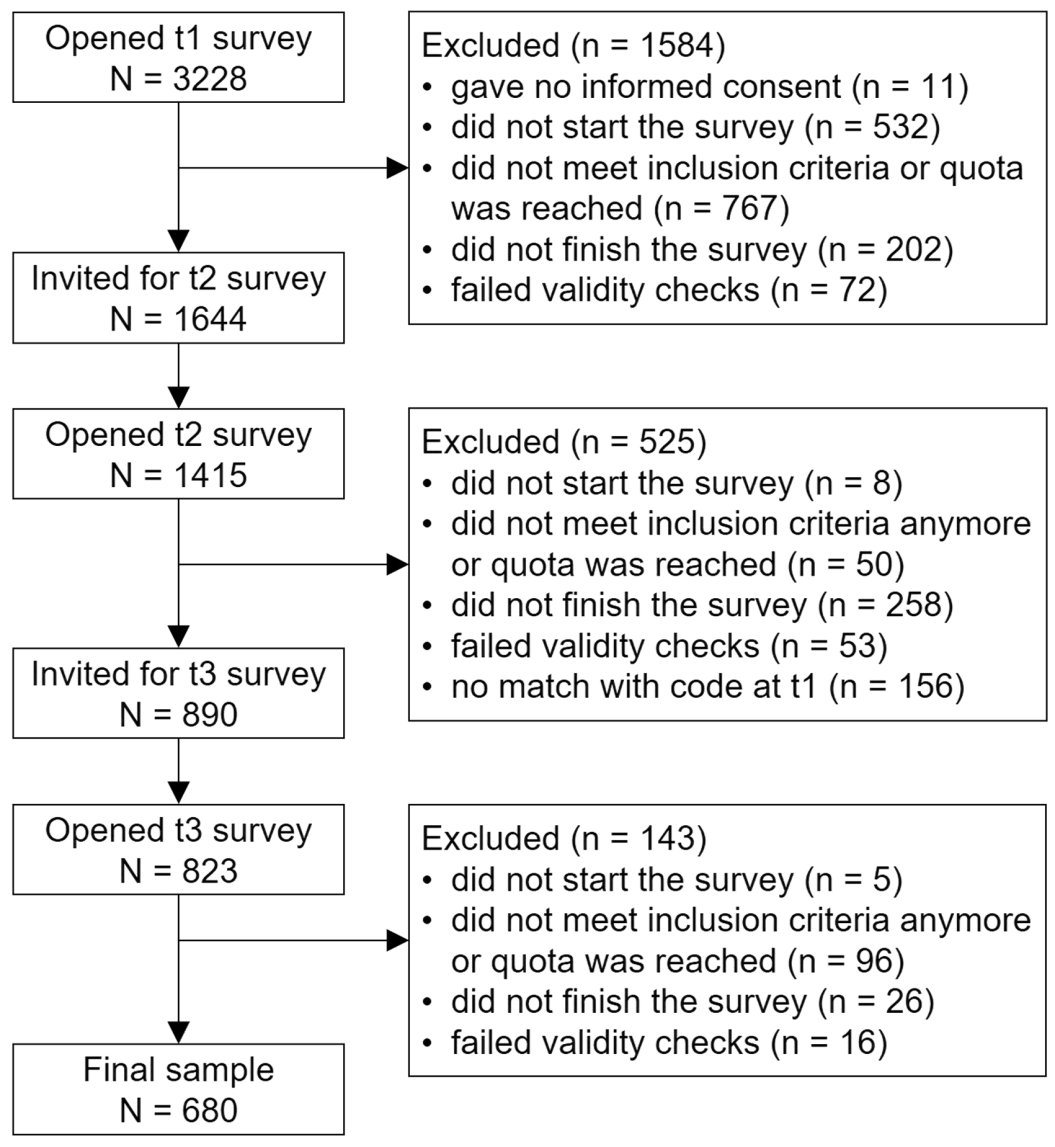
Participant flow chart.

The following inclusion criteria were used: giving informed consent, studying at one of the 13 conventional public Dutch universities, being enrolled in a bachelor or master program, following a full-time study, being not older than 30 years, and fully completing the surveys. Sampling quotas were utilized to ensure heterogeneity and representativeness of the sample regarding university, study phase, gender, and ethnicity. Several data validity checks were performed, e.g., processing time, consistency, and plausibility of answers. The participant flow is presented in Figure 1.

### Measures

The survey at the three points of measurement was administered in Dutch. The study-related experiences were assessed regarding different timespans related to the degree universities were open for on-campus teaching and learning. The timespans were “since the beginning of the academic year” at t1 (on-campus teaching and learning was possible at a low level), “since January 2021” at t2 (no on-campus teaching and learning was possible), and “since the partial re-opening of the universities in May 2021” at t3 (on-campus teaching and learning was possible at a low level).

In the following section, only measures relevant to the present study are described, i.e., measures assessing study-related wellbeing (i.e., academic burnout and study engagement), study-related behavior (i.e., study effort), and study-related attitudes (i.e., education satisfaction, online self-efficacy, and attitudes toward online education).

*Academic burnout* during the indicated timespan was measured with the emotional exhaustion subscale from the Utrecht Burnout Scale for students (UBOS-S; Schaufeli et al., 2002). The five items (e.g., “I feel used up at the end of a day at university.”) were scored on a 7-point scale ranging from 0 (never) to 6 (always). Items were averaged so that higher scores indicate greater academic burnout. Earlier research confirmed the reliability and validity of the UBOS-S (Schaufeli et al., 2002). In the present study, Cronbach’s alpha was 0.86 at t1 and 0.88 at t2 and t3.

*Study engagement* during the indicated timespan was assessed by using the ultra-short Utrecht Work Engagement Scale-Student Form (UWES-3-SF; Gusy et al., 2019). The three items (e.g., “My studies inspire me.”) reflect the dimensions vigor, dedication, and absorption. Responses were given on a 7-point scale from 0 (never) to 6 (always). Items were averaged so that higher scores indicate greater study engagement. The reliability and validity of this scale has been proven in earlier studies (Gusy et al., 2019). In the present study, Cronbach’s alpha was 0.76 at t1 and 0.80 at t2 and t3.

*Study effort* during the indicated timespan was assessed by using a shortened version of the school effort scale (Butler, 2007). The three items (e.g., “I put forth a high level of effort in class.”) were answered on a 7-point scale from 0 (never) to 6 (always) and averaged so that higher scores indicate greater effort invested in the study. In earlier studies, (adapted versions of) the school effort scale proved to be reliable and valid (Butler, 2007; van Herpen et al., 2017; van Herpen, 2019). In the present study, Cronbach’s alphas were 0.76, 0.82, and 0.83 at t1, t2, and t3, respectively. Additionally, at all measurement points, a principal component analysis suggested a one-factor solution with factor loadings of ≥0.78 for all items and an explained variance of ≥68%.

Following Sears et al. (2017), *education satisfaction* during the indicated timespan was measured with the single item “All things considered, how satisfied are you with the educational offer as a whole?.” Responses were given on a visual analog scale ranging from 0 (extremely dissatisfied) to 10 (extremely satisfied). The usefulness of single-item measures to assess domain-specific satisfaction, such as job satisfaction or life satisfaction, has been demonstrated in earlier research (Fisher et al., 2016; Jovanović and Lazić, 2020).

*Online self-efficacy* during the indicated timespan was assessed using the four items with the highest factor loadings of the scale Self-efficacy to complete an online course (Shen et al., 2013). Responses to the items (e.g., “I can complete an online course with a good grade.”) were given on a 5-point scale from 1 (strongly disagree) to 5 (strongly agree). Items were averaged so that higher scores indicate a higher level of online self-efficacy. The reliability and validity of the original scale has been proven in earlier studies (Shen et al., 2013). In the present study, Cronbach’s alpha was 0.82 at t1 and 0.86, at t2 and t3. Additionally, at all measurement points, a principal component analysis suggested a one-factor solution with factor loadings of ≥0.78 for all items and an explained variance of ≥65%.

*Attitudes toward online education* during the indicated timespan was measured using the first four items of the scale Attitudes toward ICT for learning (Ng, 2012). The items were relevant in the context of ERT (e.g., “I like online education.”) and were answered on a 5-point scale from 1 (strongly disagree) to 5 (strongly agree). Items were averaged so that higher scores indicate more positive attitudes toward online education. The reliability and validity of the original scale has been proven in earlier studies (Ng, 2012; Jeon and Kim, 2022). In the present study, Cronbach’s alphas were 0.86, 0.88, and 0.90 at t1, t2, and t3, respectively. Additionally, at all measurement points, a principal component analysis suggested a one-factor solution with factor loadings of ≥0.80 for all items and an explained variance of ≥71%.

### Data analysis

Per outcome measure (i.e., academic burnout, study engagement, study effort, education satisfaction, online self-efficacy, attitudes toward online education), a 3 (time: t1 vs. t2 vs. t3) × 2 (gender: male vs. female) × 2 (study phase: bachelor vs. master) mixed design analysis of variance (ANOVA) was conducted (Field, 2013) to investigate the change of the study-related experiences over time (main effects for time) and whether these changes were modified by gender and/or study phase (interaction effects for time × gender and time × study phase). Significant main effects for time were further analyzed using pairwise comparisons with Bonferroni adjustment. Significant interaction effects for time × gender or time × study phase were further analyzed by Bonferroni adjusted simple main effects analyses (Bibby, 2010). Significant interaction effects of gender x study phase were not further analyzed due to irrelevance regarding the research question. Eta squared (*η*^2^) is reported as effect size and classified as *η*^2^ = 0.01 as small, *η*^2^ = 0.06 as moderate and *η*^2^ = 0.14 as large (Cohen, 1988).

## Results

### Sample description

A total of 680 students participated at all three measurement points (see Figure 1). Of these, 448 (65.9%) identified as female, 230 (33.8%) identified as male, and 2 (0.3%) identified as non-binary. The two non-binary students were excluded from the statistical analyses as the group is too small to permit meaningful comparisons.

The sample used for analyses had a mean age of 21 years (*SD* = 2.06; range 17–28 years). Most students were native Dutch (87.3%), while 6.5% had a Western migration background and 6.2% had a non-Western migration background. Most of the students lived in student housing or with friends (60.5%), followed by living with their parents/family (26.3%), alone (8.7%), with their partner (3.5%), or in another form (1.0%).

Students from all 13 conventional Dutch universities and from all study fields participated (see Table 1). About two-thirds of the students (69.2%) were enrolled in a bachelor program, while the remainder (30.8%) was enrolled in a master program.

**Table 1:** Study characteristics of the analyzed sample (*N* = 678).

University	*n* (%)	Field of study	*n* (%)
Delft University of Technology	66 (9.7)	Agriculture and environment	16 (2.4)
Eindhoven University of Technology	33 (4.9)	Economics and business	86 (12.7)
Erasmus University Rotterdam	76 (11.2)	Education	8 (1.2)
Leiden University	78 (11.5)	Engineering	134 (19.8)
Maastricht University	26 (3.8)	Healthcare	102 (15.0)
Radboud University Nijmegen	35 (5.2)	Languages, arts, and culture	40 (5.9)
Tilburg University	48 (7.1)	Law	76 (11.2)
University of Amsterdam	67 (9.9)	Science and informatica	42 (6.2)
University of Groningen	68 (10.9)	Social sciences	145 (21.4)
University of Twente	23 (3.4)	Multidisciplinary	29 (4.3)
Utrecht University	78 (11.5)		
VU Amsterdam	45 (6.6)		
Wageningen University	35 (5.2)		

As regards the university affiliation, the field of study, and the study phase, the present sample is reasonably representative of the Dutch student population in the academic year 2020–2021. However, male students and students with migration background are underrepresented (DUO, 2021a; DUO, 2021b). A comparison of our sample with the student population in the Netherlands in the academic year 2020-2021 can be found in Supplementary Table S1.

### Changes in study-related experiences over time (modified by gender and study phase)

The results of the six 3 (time: t1 vs. t2 vs. t3) × 2 (gender: male vs. female) × 2 (study phase: bachelor vs. master) mixed design ANOVAs are summarized in Table 2.

**Table 2:** Results of the six 3 (time: t1 vs. t2 vs. t3) × 2 (gender: male vs. female) × 2 (study phase: bachelor vs. master) mixed design analyses of variance.

	Academic burnout		Study engagement		Study effort		Education satisfaction		Online self-efficacy		Attitudes to online education	
	*F*	*η* ^2^	*F*	*η* ^2^	*F*	*η* ^2^	*F*	*η* ^2^	*F*	*η* ^2^	*F*	*η* ^2^
Time	20.69^***^	0.03	8.21^***^	0.01	4.05^*^	0.01	26.00^***^	0.04	9.70^***^	0.01	11.04^***^	0.02
Gender	19.74^***^	0.03	3.74	0.01	1.94	0.00	0.29	0.00	1.88	0.00	0.77	0.00
Study phase	1.16	0.00	11.31^***^	0.02	21.45^***^	0.03	27.39^***^	0.04	23.04^**^	0.03	0.07	0.00
Time × gender	0.33	0.00	1.25	0.00	1.31	0.00	0.49	0.00	1.64	0.00	0.04	0.00
Time × study phase	1.19	0.00	1.05	0.00	3.00	0.00	1.20	0.00	0.03	0.00	2.94	0.00
Gender × study phase	0.61	0.00	1.33	0.00	0.02	0.00	1.70	0.00	4.62^*^	0.01	4.13^*^	0.01
Time × gender × study phase	0.98	0.00	2.39	0.00	0.53	0.00	1.35	0.00	0.58	0.00	2.92	0.00

Eta squared (*η*^2^) is reported as effect size and classified as *η*^2^ = 0.01 as small, *η*^2^ = 0.06 as moderate and *η*^2^ = 0.14 as large (Cohen, 1988). ^***^p < 0.001, ^**^p < 0.01, ^*^p < 0.05.

**Figure 2:**
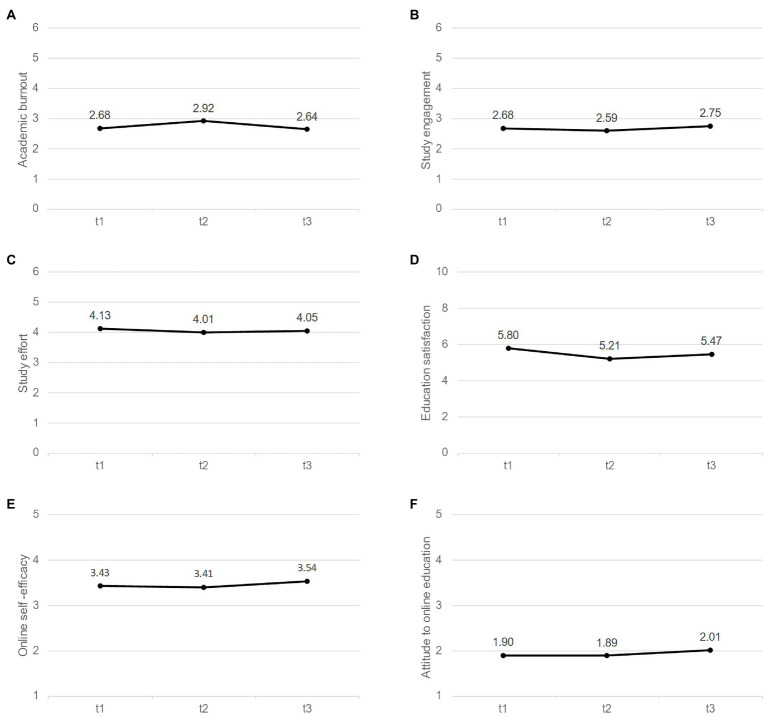
Changes over time in study-related experiences.

The ANOVA regarding *academic burnout* revealed small significant main effects for time and gender. The pairwise comparisons regarding the main effect for time (see Figure 2A) imply that burnout scores were significantly higher at t2 than at t1 and t3, *ps* < 0.001, while no significant difference was observed between t1 and t3, *p* = 1.00. The main effect for gender implies that male students (*M* = 2.53, *SE* = 0.08) had significantly lower burnout scores compared to female students (*M* = 2.96, *SE* = 0.06), *p* < 0.001.

The ANOVA regarding *study engagement* revealed small significant main effects for time and study phase. The pairwise comparisons regarding the main effect for time (see Figure 2B) imply that scores on study engagement were significantly lower at t2 than at t3, *p* < 0.001, and marginally significantly lower than at t1, *p* = 0.061, while no significant differences was observed between t1 and t3, *p* = 0.345. The main effect for study phase implies that bachelor students (*M* = 2.53, *SE* = 0.05) had significantly lower scores on study engagement compared to master students (*M* = 2.81, *SE* = 0.07), *p* < 0.001.

The ANOVA regarding *study effort* revealed small significant main effects for time and study phase. The pairwise comparisons regarding the main effect for time (see Figure 2C) imply that scores on study effort were significantly lower at t2 than at t1, *p* = 0.019. No other comparisons were significant, *p*s > 0.237. The main effect for study phase implies that bachelor students (*M* = 3.88, *SE* = 0.05) had significantly lower scores on study effort compared to master students (*M* = 4.25, *SE* = 0.07), *p* < 0.001.

The ANOVA regarding *education satisfaction* revealed small significant main effects for time and study phase. The pairwise comparisons regarding the main effect for time (see Figure 2D) implies that scores on education satisfaction significantly differed at all three points of measurement, *p*s < 0.01, with lowest scores at t2 and highest scores at t1. The main effect for study phase implies that bachelor students (*M* = 5.15, *SE* = 0.08) had significantly lower scores on education satisfaction compared to master students (*M* = 5.84, *SE* = 0.11) *p* < 0.001.

The ANOVA regarding *online self-efficacy* revealed small significant main effects for time and study phase. The pairwise comparisons regarding the main effect for time (see Figure 2E) imply that online self-efficacy was significantly higher at t3 than at t1, *p* = 0.003, and t2, *p* < 0.001, while no significant difference was observed between t1 and t2, *p* = 1.00. The main effect for study phase implies that bachelor students (*M* = 3.29, *SE* = 0.04) had significantly less online self-efficacy compared to master students (*M* = 3.62, *SE* = 0.06), *p* < 0.001.

The ANOVA regarding *attitudes toward online education* revealed a small significant main effect for time. The pairwise comparisons regarding the main effect for time (see Figure 2F) imply that scores on attitudes toward online education were significantly higher at t3 than at t1, *p* = 0.002, and t2, *p* < 0.001, while no significant difference was observed between t1 and t2, *p* = 1.00.

## Discussion

Using a longitudinal natural experiment design, we investigated whether and how study-related experiences of Dutch university students changed during the pandemic in the face of varying limitations in traditional on-campus teaching and learning. Additionally, we explored the role of gender and study phase in (the changes of) students’ study-related experiences.

Throughout the academic year 2020–2021 in which ERT was the main form of education, Dutch university students reported moderate levels of study-related wellbeing (i.e., academic burnout and study engagement), study effort, and education satisfaction. These findings are comparable to those of previous studies conducted during the pandemic (Aristovnik et al., 2020; Moreno-Fernandez et al., 2020; Aldhahi et al., 2021; Hamdan et al., 2021; Heidari et al., 2021; Lin, 2021; Silistraru et al., 2021; Simsek et al., 2021; Sveinsdóttir et al., 2021; Teuber et al., 2021; Wang et al., 2021; Lee K. et al., 2021; Azzi et al., 2022; Ertek et al., 2022; Kwan, 2022; Silistraru et al., 2022). Compared to studies conducted in the pre-pandemic period using the same measures to assess student-related wellbeing (Faye-Dumanget et al., 2017; Gusy et al., 2019; Lesener et al., 2020), students in our sample had considerably higher scores of academic burnout and considerably lower scores of study engagement. This suggests that students’ study-related wellbeing has been negatively affected by the pandemic-induced changes, which is in line with longitudinal studies showing that the pandemic had detrimental effects on students’ mental health (Bu et al., 2020; Elmer et al., 2020; Huckins et al., 2020; Li et al., 2020; Saraswathi et al., 2020; Deng et al., 2021; Fruehwirth et al., 2021; Meda et al., 2021; Savage et al., 2021; Werner et al., 2021; Zimmermann et al., 2021). It, however, contradicts longitudinal studies that did not find unfavorable changes in study-related wellbeing (Pasion et al., 2021; Schindler et al., 2021; Zis et al., 2021; Azzi et al., 2022).

As regards the changes during the pandemic, students’ study-related wellbeing, education satisfaction, and study effort declined when the measures were strengthened during the national lockdown and the partial closure of universities turned into a full closure with no on-campus teaching and learning. During this period with the highest percentage of online education, students experienced the highest levels of academic burnout and the lowest levels of study engagement and education satisfaction. After the partial re-opening of the universities at the end of the academic year, the study-related wellbeing returned to its initial levels, while education satisfaction slightly increased, but did not return to its initial level. These findings indicate that changes in the extent to which on-campus teaching and learning is possible are associated with changes in academic burnout, study engagement, and education-satisfaction. It should be noted that these changes in the study-related experiences, although statistically significant, were small in size, which might suggest that students were rather able to well handle the challenges of ERT. On the other hand, the differences in limitations of traditional on-campus teaching and learning during the pandemic were also quite small, however, these changes seem to translate into changes in study-related experiences. Our findings are in accordance with studies showing that after the initial decline after the onset of the pandemic, students’ mental health remained relatively stable at a lower level with little variations dependent on the strictness of the measures (Amendola et al., 2021; Meda et al., 2021; Weber et al., 2022).

Overall, Dutch university students had moderate levels of online self-efficacy and negative attitudes toward online education, which is in line with earlier research conducted during the pandemic (Aldhahi et al., 2021; Barzani and Jamil, 2021; Guven Ozdemir and Sonmez, 2021; Hamdan et al., 2021; Lin, 2021; Talsma et al., 2021; Geçer and Bağci, 2022). Both students’ trust in their ability to successfully complete an online course and attitudes toward online education increased at the end of the academic year, indicating that after a year of limitations in on-campus teaching and learning, students might have gotten familiar with online education (Zhu et al., 2020). However, this increase has a small effect size and, although increased, the attitudes toward online education were still negative. This is not surprising as the students in the present sample chose to study at a conventional university in which on-campus teaching and learning is the norm instead of an open or distance university in which all courses are offered online or in hybrid form (Sankaran et al., 2000; Benson, 2005; Garcia et al., 2013; Fidalgo et al., 2020).

The role of gender is negligible as no differences in the changes of study-related experiences were found between male and female students. This suggests that male and female students were equally affected by the limitations in on-campus teaching and learning. One overall difference indicates that female students experienced more academic burnout than male students, which supports findings of studies conducted in the pre-pandemic period (Worly et al., 2019; Rosales-Ricardo et al., 2021).

Also, no effect of study phase on the changes of study-related experiences was found, indicating that the limitations in on-campus teaching and learning equally affected bachelor and master students. However, bachelor students reported overall less study engagement, less education satisfaction, less study effort, and less online self-efficacy than master students. This is in line with few previous studies conducted during the pandemic (Aristovnik et al., 2020; Simsek et al., 2021) and leads to the conclusion that bachelor students have in general more negative study-related experiences.

### Practical implications

Since high levels of online education impair students’ study-related wellbeing, education satisfaction, and study effort, policymakers and universities should carefully consider the balance between students’ experiences and public health concerns when determining the (partial) closure of university campuses in future waves of COVID-19 or other pandemics. Our findings imply that universities should (be allowed to) provide and facilitate on-campus teaching and learning as much and as long as possible to preserve students’ study-related wellbeing, education satisfaction, and study effort. This might be beneficial to students’ academic performances and achievements (Upadyaya and Salmela-Aro, 2013; Chang et al., 2014; Schneider and Preckel, 2017; Lei et al., 2018; Madigan and Curran, 2020; Pu et al., 2020; Gopal et al., 2021).

However, when on-campus education must be restricted or prohibited due to severe public health concerns, online education should be implemented in a way that students’ study-related wellbeing, education satisfaction, and study effort is preserved or even facilitated. Previous research showed that meaningful and supportive student-teacher and student-peer relationships are crucial for positive study-related experiences (Lee M. et al., 2021; Salmela-Aro et al., 2022; Sun et al., 2022; van Herpen et al., 2022; Versteeg et al., 2022; Wissing et al., 2022). Consequently, especially during times of online education, opportunities for students to frequently interact with teachers and peers should be implemented. For example, during synchronous course activities, e.g., tutorials *via* video conferencing platforms, active learning techniques such as flipped classroom and group discussion as well as interactive tools such as chat and raise hand can be used (Morgan and Chen, 2021; Rossi et al., 2021; Muir et al., 2022). Additionally, social interaction and student engagement can be stimulated by implementing discussion boards, (peer) feedback, and group assignments (Baran and AlZoubi, 2020; Majewska and Vereen, 2021; Muir et al., 2022). Finally, more informal student-teacher and student-peer interaction can be facilitated by setting up online (peer) mentoring programs or a student-led peer support service (Pollard and Kumar, 2021; Suresh et al., 2021; Krause and Moore, 2022).

### Strengths, limitations and future directions

This study provides unique insights into students’ study-related experiences during the pandemic. One key aspect is the longitudinal natural experiment design. This design allowed us to examine changes in study-related experiences over time dependent on the degree of limitations in traditional on-campus teaching and learning. Another strength is the heterogeneity and largely representativity of the sample which consists of students from all conventional Dutch universities, study fields, and study phases. This contributes to the external validity and generalizability of the findings.

However, some limitations of the present study need to be acknowledged. First, based on the strictness of the COVID-19 measures that were imposed by the Dutch government and based on the reported average percentage of online education, we argue that limitations in traditional on-campus teaching and learning varied between the three measurement points. However, this might not be true for all students. Some study programs might have refrained from offering on-campus teaching at the beginning and the end of the academic year. Additionally, some students, e.g., students at higher risk of severe COVID-19 due to an underlying health condition and students with a long travel distance, might have refrained from the possibilities of on-campus learning at the beginning and the end of the academic year. This might have reduced the overall magnitude of changes in students’ study-related experiences. Second, across the three measurement points, not only limitations in on-campus teaching and learning varied, but also restrictions in everyday life, which might also have had an impact on study-related experiences through stress contagion and spillover effects (Liu and Doan, 2020; Dey, 2021; Hong et al., 2021). Finally, potential limitations regarding the sample should be mentioned. Although the sample was fairly representative of the Dutch student population with respect to university, study field, and study phases, male students and students with migration background were underrepresented. Moreover, due to the convenience sampling method, information on response rate and non-responder characteristics is unavailable. Additionally, there might be a selection bias toward highly resilient and motivated students.

The present findings indicate that study-related wellbeing, study effort, and education satisfaction changed over time dependent on the degree of limitations in traditional on-campus teaching and learning. Future research should focus on systematically investigating study-related environmental (e.g., university facilities and services) and personal (e.g., study motivation or learning styles) factors that might buffer or even mitigate the negative effects of online education on students’ experiences. Subsequently, these protective factors could be addressed in future periods of forced online teaching. Another interesting topic for future research would be exploring the interrelations between the study-related experiences and the long-term effects of ERT on academic performance. As attitudes toward online education have been found to be rather negative, it would also be valuable to investigate determinants of attitudes toward online education and how these could be targeted. Finally, although a wide range of study-related experiences has been examined in the present study, future research could include more study-related experiences (e.g., all components of academic burnout, study-related self-efficacy, and online self-direction) to get a more comprehensive picture of the effects of the pandemic-induced changes in university education on students’ experiences.

### Conclusion

Overall, students reported moderate levels of study-related wellbeing, study effort, education satisfaction, and online self-efficacy. During the pandemic, students’ study-related wellbeing was impaired compared to the pre-pandemic period (Faye-Dumanget et al., 2017; Gusy et al., 2019; Lesener et al., 2020). Additionally, students’ study-related wellbeing and education satisfaction slightly varied dependent on the slight changes in limitations in on-campus teaching and learning. The findings imply that students were better off in periods in which on-campus teaching and learning was possible even though it was at a low level and with several restrictions compared to the period in which the campuses were fully closed. Consequently, to preserve students’ study-related wellbeing, education satisfaction, study effort, and finally student’s academic success, universities should (be allowed to) open their campuses and facilitate on-campus teaching and learning as much as possible in times of pandemics.

## Data availability statement

The datasets presented in this study can be found in online repositories. The names of the repository/repositories and accession number (s) can be found at: Erasmus University Rotterdam’s data repository; https://doi.org/10.25397/eur.21155347.

## Ethics statement

The studies involving human participants were reviewed and approved by the Medical Ethics Review Committee of the Erasmus Medical Center (#2020–0815). The patients/participants provided their written informed consent to participate in this study.

## Author contributions

MV and FH organized the data collection and database. MV performed the statistical analysis and wrote the first draft of the manuscript. All authors contributed to the conception and design of the study, manuscript revision, read, and approved the submitted version.

## Funding

This research was supported by a grant provided by the Netherlands Organisation for Health Research and Development (ZonMw; grant no. 10430 03201 0023).

## Conflict of interest

The authors declare that the research was conducted in the absence of any commercial or financial relationships that could be construed as a potential conflict of interest.

## Publisher’s note

All claims expressed in this article are solely those of the authors and do not necessarily represent those of their affiliated organizations, or those of the publisher, the editors and the reviewers. Any product that may be evaluated in this article, or claim that may be made by its manufacturer, is not guaranteed or endorsed by the publisher.
