# Supplementary material for: Study-related wellbeing, behavior, and attitudes of university students in the Netherlands during emergency remote teaching in the context of COVID-19: A longitudinal study
Source: Front Psychol. 2022 Dec 6;13:1056983. doi: 10.3389/fpsyg.2022.1056983 (PMC9764013; doi:10.3389/fpsyg.2022.1056983)
Supplement: Supplementary file 1 [file Table_1.pdf]

Vollmann, M., Scheepers, R. A., Nieboer, A. P., and Hilverda, F. (2022). Study-related wellbeing, behavior, and attitudes of university students in the Netherlands during emergency remote teaching in the context of COVID-19: A longitudinal study. Front. Psychol. 13:1056983. doi: 10.3389/fpsyg.2022.1056983

Table S1: Characteristics of the present sample and the student population in the Netherlands in the academic year 2020–2021

	**Sample (*N* = 680)**	**Student population x 1.000[a]**
**Gender**		
Male	230 (33.8%)	155.4 (47.2%)
Female	448 (65.9%)	173.6 (52.8%)
Non-binary	2 (0.3%)	-
**Migration background**		
Yes	87 (12.8%)	128.8 (39.1%)
No	593 (87.2%)	200.2 (60.9%)
**University**		
Delft University of Technology	66 (9.7%)	6.0 (7.9%)
Eindhoven University of Technology	33 (4.9%)	12.9 (3.9%)
Erasmus University Rotterdam	76 (11.2%)	30.1 (9.1%)
Leiden University	78 (11.5%)	32.4 (9.9%)
Maastricht University	26 (3.8%)	19.7 (6.0%)
Radboud University Nijmegen	35 (5.1%)	23.6 (7.2%)
Tilburg University	48 (7.1%)	19.3 (5.9%)
University of Amsterdam	69 (10.1%)	39.0 (11.8%)
University of Groningen	68 (10.0%)	34.1 (10.4%)
University of Twente	23 (3.4%)	12.2 (3.7%)
Utrecht University	78 (11.5%)	35.3 (10.7%)
VU Amsterdam	45 (6.6%)	29.8 (9.1%)
Wageningen University	35 (5.1%)	12.9 (3.9%)
Other universities, such as private or philosophical institutions	-	1.7 (<1%)
**Field of study**		
Agriculture and environment	16 (2.4%)	12.6 (3.8%)
Economics and business	86 (12.6)	51.3 (15.6%)
Education	8 (1.2%)	1.6 (<1%)
Engineering	134 (19.7%)	47.4 (14.4%)
Healthcare	102 (15.0%)	36.7 (11.2%)
Languages, arts, and culture	41 (6.0%)	34.4 (10.5%)
Law	76 (11.2%)	33.9 (10.3%)
Science and informatica	42 (6.2%)	37.3 (11.3%)
Social sciences	146 (21.5%)	60.3 (18.3%)
Multidisciplinary	29 (4.3%)	13.5 (4.1%)
**Study phase**		
Bachelor	470 (69.1%)	206.9 (62.9%)
Master	210 (30.9%)	120.6 (36.6%)
Premaster	-	1.5 (<1%)
**Study type**		
Fulltime	680 (100%)	323.6 (98.4%)
Parttime	-	4.8 (1.5%)
Dual	-	0.6 (<1%)

Note: [a] *N* = 329,024 university students in the Netherlands enlisted on October 1st, 2020 (DUO, 2021a; DUO, 2021b).
